# Selective inhibition of N-linked glycosylation impairs receptor tyrosine kinase processing

**DOI:** 10.1242/dmm.039602

**Published:** 2019-06-05

**Authors:** Elsenoor Klaver, Peng Zhao, Melanie May, Heather Flanagan-Steet, Hudson H. Freeze, Reid Gilmore, Lance Wells, Joseph Contessa, Richard Steet

**Affiliations:** 1 Complex Carbohydrate Research Center, University of Georgia, Athens, GA 30602, USA; 2 Research Division, Greenwood Genetic Center, Greenwood, SC 29646, USA; 3 Sanford Children's Health Research Center, Sanford-Burnham-Prebys Discovery Institute, La Jolla, CA 92037, USA; 4 Department of Biochemistry and Molecular Pharmacology, University of Massachusetts Medical School, Worchester, MA 01655, USA; 5 Departments of Therapeutic Radiology and Pharmacology, Yale University, New Haven, CT 06520, USA

**Keywords:** Congenital, Convertase, Glycoproteins, Oligosaccharyltransferase, Furin, PCSK5, INSR, IGF-1R, Protein processing, STT3B

## Abstract

Global inhibition of N-linked glycosylation broadly reduces glycan occupancy on glycoproteins, but identifying how this inhibition functionally impacts specific glycoproteins is challenging. This limits our understanding of pathogenesis in the congenital disorders of glycosylation (CDG). We used selective exo-enzymatic labeling of cells deficient in the two catalytic subunits of oligosaccharyltransferase – STT3A and STT3B – to monitor the presence and glycosylation status of cell surface glycoproteins. We show reduced abundance of two canonical tyrosine receptor kinases – the insulin receptor and insulin-like growth factor 1 receptor (IGF-1R) – at the cell surface in *STT3A*-null cells, due to decreased N-linked glycan site occupancy and proteolytic processing in combination with increased endoplasmic reticulum localization. Providing cDNA for Golgi-resident proprotein convertase subtilisin/kexin type 5a (PCSK5a) and furin cDNA to wild-type and mutant cells produced under-glycosylated forms of PCSK5a, but not furin, in cells lacking STT3A. Reduced glycosylation of PCSK5a in *STT3A*-null cells or cells treated with the oligosaccharyltransferase inhibitor NGI-1 corresponded with failure to rescue receptor processing, implying that alterations in the glycosylation of this convertase have functional consequences. Collectively, our findings show that STT3A-dependent inhibition of N-linked glycosylation on receptor tyrosine kinases and their convertases combines to impair receptor processing and surface localization. These results provide new insight into CDG pathogenesis and highlight how the surface abundance of some glycoproteins can be dually impacted by abnormal glycosylation.

## Abstract (precis)

**Summary:** Selective reduction in N-glycan occupancy impacts the processing of two receptor tyrosine kinases by increasing their ER localization and compromising the activity of the convertases responsible for their maturation.

## INTRODUCTION

Glycosylation on cell surface receptors regulates their activity, stability and interaction with ligands (Rudd et al., 2001; Shao and Haltiwanger, 2003; Boscher et al., 2011; Takahashi et al., 2016). Numerous studies have demonstrated the direct effects of specific glycosyltransferases on receptor function in the context of common disease states, including cancer and diabetes (Ohtsubo and Marth, 2006). In contrast, much less is known about how global defects in glycosylation, such as those found in many congenital disorders of glycosylation (CDG), alter the maturation, stability and function of receptors and other cell surface glycoproteins. Defining these effects will provide clues towards the complex and multisystem pathogenesis of CDG and has added value in understanding how small-molecule modulation of glycosylation might be harnessed for the treatment of more common diseases.

Asparagine-linked glycosylation is initiated through the biosynthesis of a 14-sugar lipid-linked oligosaccharide (LLO) precursor on the cytosolic and luminal sides of the endoplasmic reticulum (ER). The ER-localized oligosaccharyltransferase (OST) complex is responsible for the transfer of the LLO precursor to nascent polypeptides in the ER lumen (Kelleher and Gilmore, 2006). The two metazoan OST complexes contain one of two catalytic subunits, STT3A or STT3B, five shared subunits plus complex-specific accessory subunits that impact glycan-acceptor-site recognition (Kelleher et al., 2003; Cherepanova et al., 2016). STT3A has been demonstrated to carry out the co-translational N-linked glycosylation of nascent proteins as they enter the ER lumen, whereas STT3B is responsible for post-translational N-linked glycosylation of substrates, including the transfer of oligosaccharide to extreme C-terminal sites and to regions proximal to disulfide bridges (Ruiz-Canada et al., 2009; Shrimal and Gilmore, 2013; Shrimal et al., 2013b). Two new CDG types caused by mutations in STT3A and STT3B were described recently, indicating that optimal function of both subunits is required in humans despite compensation in their function on a cellular level (Shrimal et al., 2013a; Cherepanova and Gilmore, 2016).

Prior studies using an inhibitor of STT3 function (NGI-1) demonstrated that receptor tyrosine kinases such as epidermal growth factor receptor (EGFR) are strongly sensitive to blockade in the N-glycosylation pathway, reducing cell surface localization and signaling of the EGFR glycoprotein, and selectively arresting proliferation in cell lines that are dependent on EGFR for survival (Contessa et al., 2008; Lopez-Sambrooks et al., 2016; Baro et al., 2019). As all receptor tyrosine kinases are N-glycosylated, it is likely that loss of OST function will functionally impact other receptors in addition to EGFR, as well as different cell surface glycoproteins. Impaired N-linked glycosylation can in theory directly impact receptors on many levels, including their folding and cell surface stability, but also by indirectly impacting the enzymes and proteins involved in the proteolytic maturation and recycling of the receptors. Processing of many receptors is carried out by proprotein convertases, such as furin and PCSK5. These endoproteases typically reside in the trans-Golgi network and convert large inactive receptors into their bioactive mature forms.

Using selective exo-enzymatic labeling (SEEL) of cells deficient in each of the OST catalytic subunits, we identified reduced abundance of several glycoproteins, including the insulin receptor (INSR) and IGF-1R, at the cell surface in *STT3A*-null cells. The reduction of these receptors at the cell surface corresponded with decreases in the occupancy of their N-linked glycans and ER localization, and a drastic reduction in their proteolytic maturation. We further identified the proprotein convertase PCSK5 as another STT3A substrate and show that overexpression of furin but not PCSK5a in *STT3A*-null cells is capable of rescuing the impaired processing of both INSR and IGF-1R. Acute inhibition of N-glycosylation using NGI-1 recapitulated the receptor glycosylation and processing defects, and indicated that the functional consequences of this pharmacological inhibition may extend to the function of both PCSK5a and furin. These findings uncover a mechanism whereby STT3A-specific defects in the N-glycosylation of receptor tyrosine kinases and certain convertases that process them combine to limit cell surface availability of the mature receptors. The implications of this discovery towards CDG pathogenesis and the use of N-glycosylation inhibitors for cancer therapy are considered.

## RESULTS

### SEEL-based proteomics identifies reduced cell surface expression of receptor tyrosine kinases in *STT3A*-null cells

**Fig. 1.**
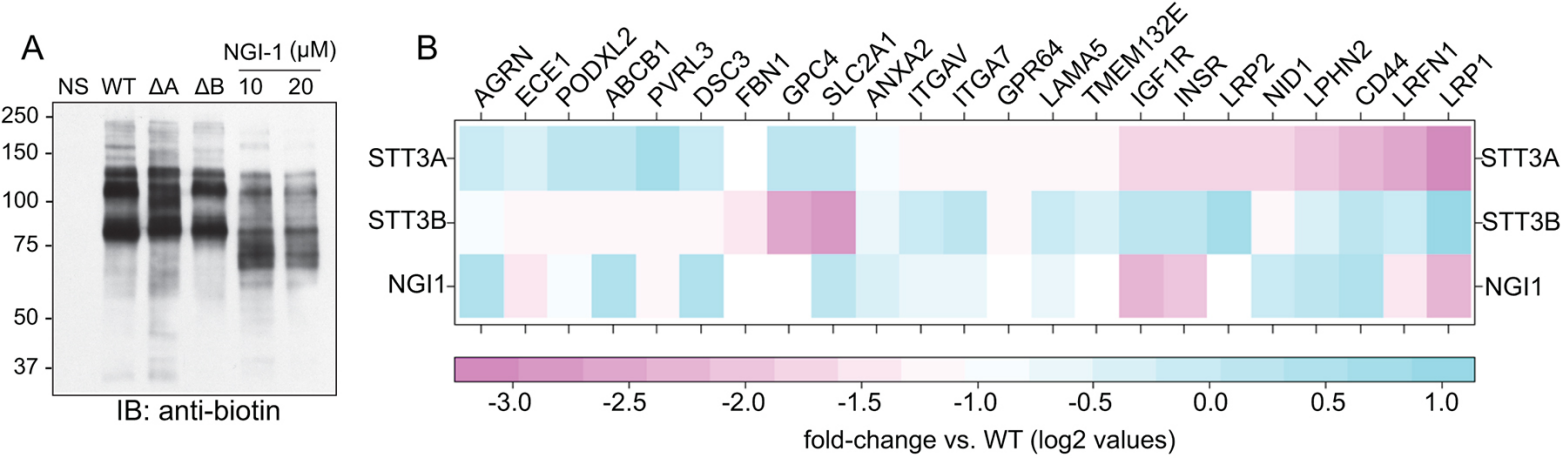
**SEEL-based proteomics identifies reduced cell surface expression of receptor tyrosine kinases in *STT3A*-null cells.** (A) Representative western blot using an anti-biotin antibody from four biological replicates of biotin-labeled glycoproteins following SEEL with ST6Gal1 in WT, *STT3A*-null (ΔA), *STT3B*-null (ΔB) and 10 µM NGI-1-treated HEK cells. (B) Heat map overview of all glycoproteins with 2-fold or more reduction in abundance after SEEL proteomics in *STT3A*-null, *STT3B*-null and 10 µM NGI-1-treated HEK cells compared to WT. Average of two proteomics runs.

Our prior work established SEEL-based proteomics as a highly effective way to assess cell surface glycoprotein abundance in cells (Sun et al., 2016; Yu et al., 2016). Wild-type (WT), *STT3A*-null and *STT3B*-null human embryonic kidney (HEK) cells were subjected to SEEL-based proteomics using ST6Gal1 in order to investigate how selective loss of N-glycan occupancy affects the abundance of cell surface glycoproteins. Unlike complementary approaches such as SILAC (stable isotope labeling by/with amino acids in cell culture)-based proteomics to gauge N-glycan site occupancy, SEEL-based proteomics has the added advantage of specifically tracking the abundance of glycoproteins at the plasma membrane in light of the membrane impermeability of both the sialyltransferase and the biotin-modified nucleotide-sugar donor. As shown in Fig. 1A, SEEL using ST6Gal1 labels several major glycoproteins in WT HEK cells, some of which are shifted lower in *STT3A*-null cells. No obvious effects on mobility were detected among the major labeled glycoproteins in *STT3B*-null cells. WT HEK cells were also treated with the inhibitor NGI-1 to gauge the effects of acute blockade of OST function. Nearly all the major labeled glycoproteins in NGI-1-treated cells were shifted down, demonstrating more robust effects on these proteins when OST function is acutely inhibited. This likely reflects the ability of NGI-1 to target both STT3A and STT3B (Lopez-Sambrooks et al., 2016). Following SEEL and immunoprecipitation, labeled glycoproteins were subjected to proteomic analysis and assigned proteins quantified by spectral counts in two separate runs (Table S1; only the 10 µM NGI-1 treatment was used for quantification in the two runs). The abundance of multiple glycoproteins was altered in the mutant and NGI-treated cells, including that of several receptors, cell adhesion molecules and transporters (Fig. 1B). Most notably in the STT3B mutants was SLC2A1, or the glucose transporter GLUT1 that bears a single N-glycan. Among the most affected receptors in the *STT3A*-null cells were the receptor tyrosine kinases INSR and IGF-1R. The levels of both receptors were also strongly reduced at the cell surface of NGI-1-treated HEK cells. We chose to focus on INSR and IGF-1R in light of the isoform specificity on cell surface localization and our prior work on the effects of N-glycosylation inhibition on this class of receptors.

### INSR and IGF-1R exhibit decreases in N-glycan site occupancy and greatly reduce proteolytic processing in *STT3A*-null cells

**Fig. 2.**
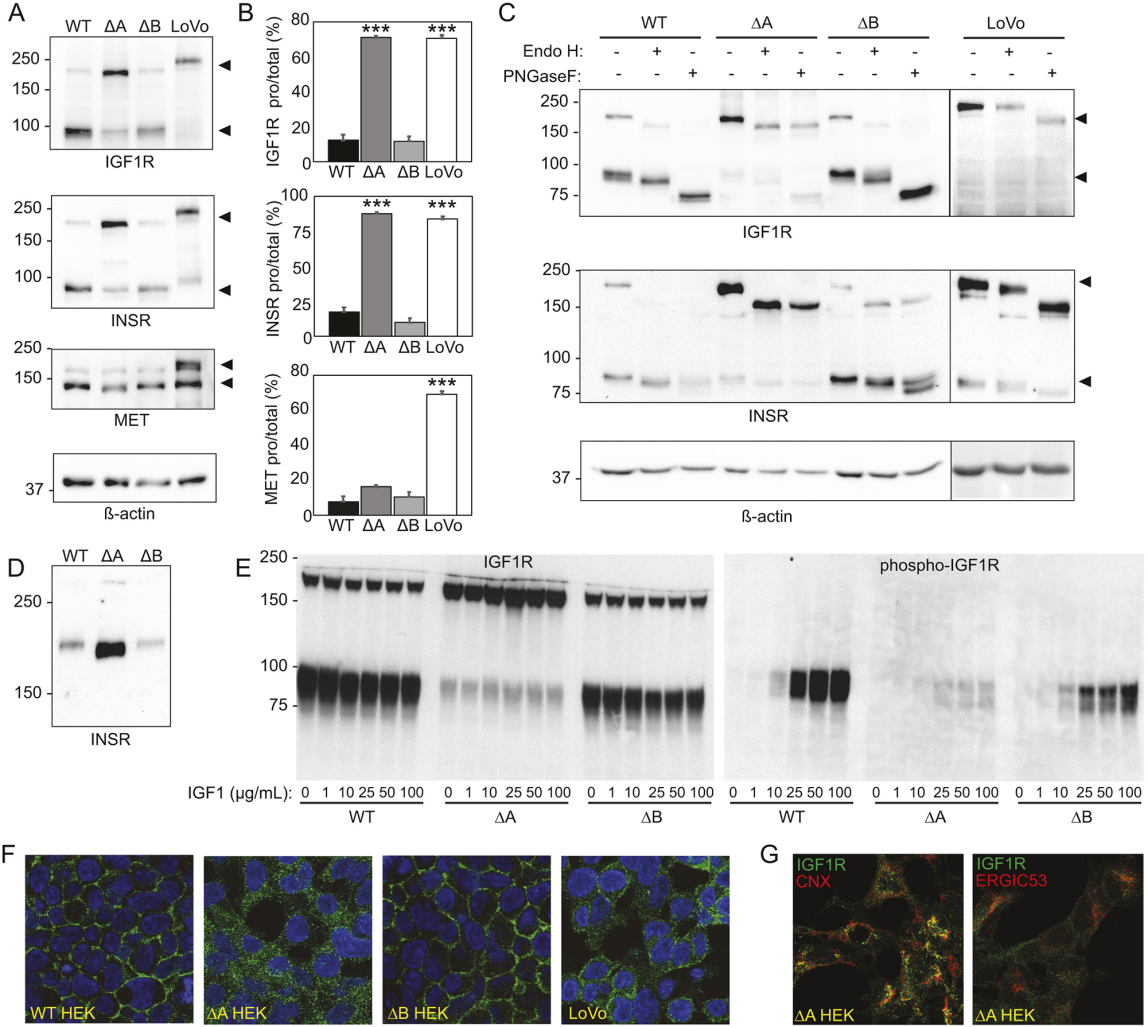
**INSR and IGF-1R exhibit modestly decreased N-glycan occupancy but greatly reduced proteolytic processing in *STT3A*-null cells.** (A) Western blot analysis of IGF-1R, INSR and Met receptor in WT, *STT3A*-null (ΔA), *STT3B*-null (ΔB) and LoVo cells. β-actin was used a loading control. Blots are representative of four individual experiments. (B) Quantification of the level of pro-receptor relative to the total amount of receptor (pro-receptor+mature receptor). Average ratios from four separate experiments are shown; error bars represent s.e.m. One-way ANOVA analysis was used to determine statistical significance. ***P<0.001. (C) Western blot analysis of IGF-1R and INSR in untreated, Endo-H_f_- and PNGase-F-treated cell lysates. (D) Western blot analysis of pro-INSR in HEK WT, *STT3A*-null and *STT3B*-null cells. (E) Analysis of IGF-1R processing and phosphorylation following IGF-1 stimulation in HEK WT, *STT3A*-null and *STT3B*-null cells. (F) IGF-1R (green) and DAPI (blue) immunostaining of HEK WT, *STT3A*-null, *STT3B*-null and LoVo cells. (G) Co-staining of IGF-1R with calnexin (CNX) and ERGIC-53 in *STT3A*-null cells.

A reduction in SEEL labeling has two possible explanations: (1) cell surface expression of the protein is lower, or (2) the protein now lacks glycans that can be modified by the SEEL procedure. The reduced level of these receptors at the cell surface could arise due to decreased expression of the receptors, impaired folding or transport due to loss of N-glycans, or other indirect effects on enzymes or proteins that process their proprotein forms to mature receptor subunits. We investigated these potential mechanisms by first performing western blotting for INSR and IGF-1R in WT, *STT3A*-null and *STT3B*-null cells (Fig. 2A). Loss of N-glycans on the receptors can be estimated by increased electrophoretic mobility of the proteins, and any effects on receptor processing are gauged by quantifying the relative level of pro-receptor and mature subunit present. In both WT HEK and *STT3B*-null cells, both receptors are largely present in the mature form (Fig. 2A). By contrast, *STT3A*-null cells show greatly reduced proteolytic processing of these receptors, with the majority of the protein (IGF-1R 71±6.9%, INSR 87.6±1.0%; means±s.e.m.) remaining in the proprotein form (Fig. 2B). A shift in molecular mass was also observed for these receptors in *STT3A*-null cells, suggestive of the loss of some of their N-glycans. A receptor processing defect in IGF-1R and INSR was observed in LoVo cells, a cancer cell line reported to lack expression of the proprotein convertase furin, although no obvious decreases in the occupancy of N-glycans was noted in these cells. Unlike either of the STT3 mutants, LoVo cells also exhibit a decrease in the processing of the Met receptor.

The pro-forms of IGF-1R and INSR localize to the ER before they move to the Golgi upon successful folding and are processed by proprotein convertases that reside in the trans-Golgi network, such as furin (Forsayeth et al., 1986; Bass et al., 1998). Lysates were treated in the absence or presence of the glycosidases Endo H and PNGase F to determine whether these receptors transited through the Golgi (the N-glycans would be Endo-H resistant) or still resided in the ER (the N-glycans would be Endo-H sensitive) (Fig. 2C). These results demonstrated that the pro-form of both receptors was Endo-H sensitive in the *STT3A*-null cells, indicative of a pre-Golgi/ER localization, whereas the mature subunits predominantly seen in WT and *STT3B*-null cells had N-glycans that were mostly Endo-H resistant, consistent with transport through the cis-Golgi and processing of the N-glycans to complex-type structures. To gain a clearer picture of the extent of reduced glycosylation on INSR, lysates were resolved on a 6% gel, providing more-definitive evidence of the loss of multiple N-glycans (Fig. 2D). Next, we looked at whether the lack of processing corresponded with a decrease in the ability of IGF-1R to bind ligand and autophosphorylate its β-subunit. WT and mutant HEK293 were stimulated with recombinant IGF1 and western blot analysis using an anti-phospho-IGF-1R antibody was performed (Fig. 2E). These data clearly show a reduction in IGF-1R activation in the *STT3A*-null cells that corresponds with the impairment in its processing. Lastly, we performed immunostaining with an antibody to IGF-1R in all four lines, confirming the intracellular localization of this receptor in *STT3A*-null cells and the surface localization in the other two lines (Fig. 2F). Co-staining experiments with IGF-1R and markers of the ER (calnexin) and ERGIC (ERGIC-53) in the *STT3A*-null cells revealed partial but not complete overlap with calnexin and only slight overlap with ERGIC-53. Staining that did not correspond with either marker was also observed, suggesting that the unprocessed receptor is likely distributed in multiple pre-Golgi compartments in these cells (Fig. 2G). These collective findings indicate that the glycosylation and proteolytic processing of both INSR and IGF-1R is impaired in *STT3A*-null cells due in part to failure of these receptors to exit the ER. The lack of processing in the *STT3A*-null cell is consistent with that observed in convertase-deficient LoVo cells (Dubois et al., 1995; Lehmann et al., 1998; Lissitzky et al., 2000). However, in LoVo cells, the receptor pro-forms are still able to escape the ER and travel to the Golgi, as evidenced by the Endo-H resistance of their N-glycans in this cell line (Fig. 2C) and partial cell surface localization of IGF-1R (Fig. 2F).

### Furin-like proprotein convertases process INSR and IGF-1R in HEK cells

**Fig. 3.**
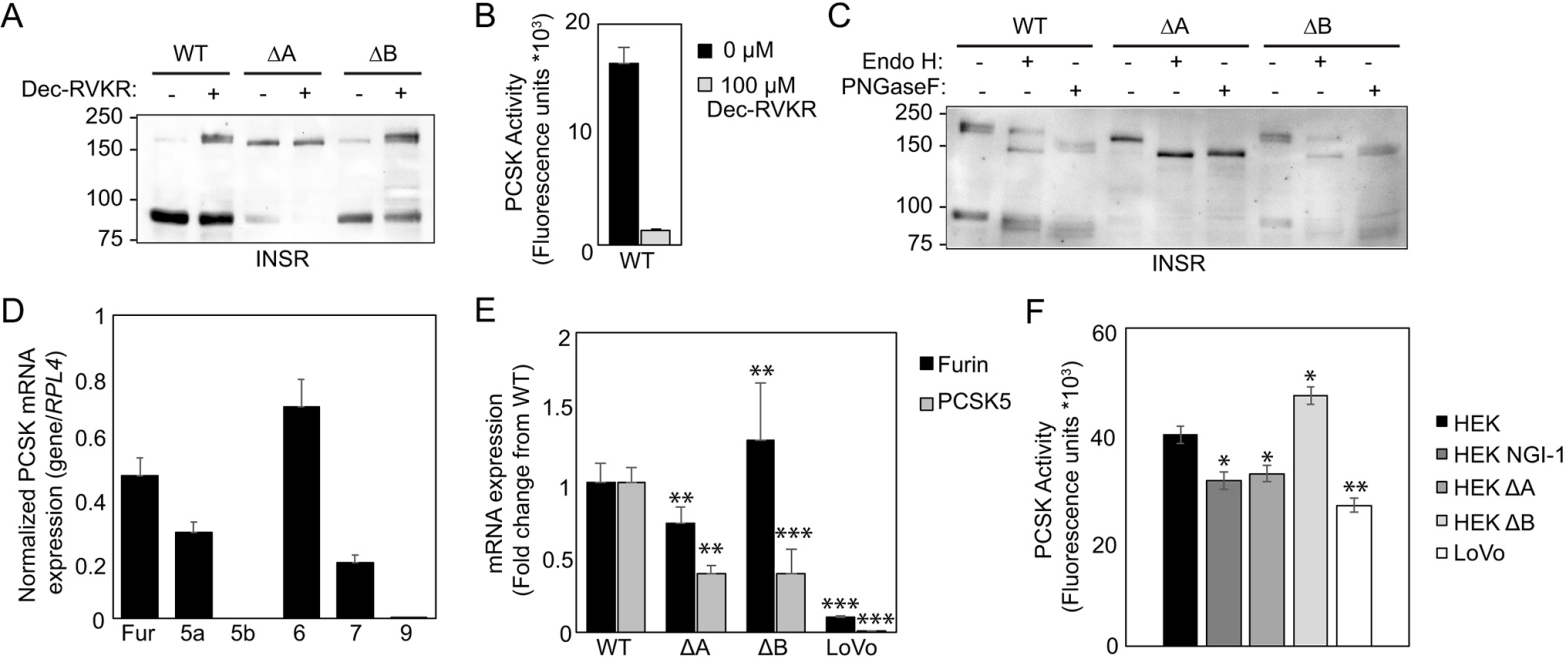
**Proprotein expression and activity are decreased in *STT3A*-null cells.** (A) INSR western blot analysis of HEK WT, *STT3A*-null (ΔA) and *STT3B*-null (ΔB) cells after treatment with PCSK inhibitor Dec-RVKR. (B) PCSK activity in HEK WT cells, with and without addition of Dec-RVKR. (C) Western blot analysis of INSR in Dec-RVKR-treated cells, followed by Endo-Hf- or PNGase-F treatment of the cell lysates. (D) Quantitative PCR analysis of PCSKs in HEK cells. (E) Quantitative PCR analysis of the transcript abundance of *PCSK5a* and furin in the different cell lines studied (average of technical duplicates from three biological replicates). One-way ANOVA analysis was used to determine statistical significance. (F) Average total convertase activity of three biological replicates towards a furin substrate in WT, mutant and NGI-1-treated HEK cells and in LoVo cells. One-way ANOVA analysis was done to determine statistical significance. Error bars represent s.e.m. *P<0.05, **P<0.01, ***P<0.001.

The reduction in receptor processing in the *STT3A*-null cells could arise solely from failure of the receptors to escape the ER, as has been demonstrated by earlier studies that show the function of specific glycans in receptor folding and transport (Collier et al., 1993; Bass et al., 1998; Hwang and Frost, 1999; Hwang et al., 2000). Reduced processing can also arise due to a decrease in the activity of the proprotein convertases that carry out receptor maturation within the secretory apparatus. We first confirmed whether furin-like convertases were responsible for processing INSR in WT and mutant HEK cells by treating these cells with a general furin inhibitor (Dec-RVKR) and determining the effects on receptor processing. As shown in Fig. 3A, INSR processing was reduced in WT and *STT3B*-null cells. The lack of a complete block likely reflects the lower dose of inhibitor we used to avoid toxicity and attachment problems seen at higher doses. The inhibitor had only a minor effect in *STT3A*-null cells since the receptor is already largely unprocessed. At concentrations used in this experiment, furin-like convertase activity was strongly lowered by the inhibitor (Fig. 3B), supporting a major role for these convertases in receptor processing in HEK cells. Next, we investigated whether the glycans of pro-form INSR were characteristic of Golgi modifications by treating with Dec-RVKR and investigating the Endo-H sensitivity of INSR (Fig. 3C). Endo-H treatment of WT and *STT3B*-null cells demonstrated that some but not all of the receptor is sensitive to this glycosidase, indicating that some of the receptor preform can still escape the ER upon convertase inhibition. In contrast, the entire population of INSR was Endo-H sensitive in *STT3A*-null cells, supporting its primary residence in the ER in Dec-RVKR-treated cells. Transcript analysis on several proprotein convertases was performed to determine which enzymes are most abundantly expressed in HEK cells (Fig. 3D). These results show that furin, PCSK5a, PCSK6 and PCSK7 are expressed in WT cells, but PCSK5b and PCSK9 are not. Two of the expressed convertases – furin and PCSK5a – are known to play a role in receptor tyrosine kinase maturation (Dubois et al., 1995; Lehmann et al., 1998; Logeat et al., 1998; Siegfried et al., 2003; Essalmani et al., 2008; Susan-Resiga et al., 2011; Al Rifai et al., 2017; Szumska et al., 2017), so we analyzed the transcript abundance of these enzymes in the STT3 mutant HEK lines and LoVo cells. Transcripts for both proteases were detected in the STT3 mutants (Fig. 3E). Transcript abundance for both proteases was much lower or absent in LoVo cells, consistent with the established loss of this convertase activity in these cells (Lehmann et al., 1998). *PCSK5* transcript abundance was decreased in both STT3 HEK mutants. To investigate whether the decreased expression corresponds with lower convertase activity, global convertase activity was measured in cell lysates using a furin substrate (Fig. 3F). The overall convertase activity in *STT3B*-null cells was higher to WT HEK cells but lower in both *STT3A*-null and NGI-1-treated HEK cells, suggesting that a reduction in N-glycan occupancy by genetic or pharmacological methods alters global convertase activity. The activity measured was mostly due to furin-like convertases as the presence of a general furin inhibitor was sufficient to inhibit over 95% of the activity detected in all lysates (not shown). Global convertase activity in LoVo cells was also decreased, to a similar level found in *STT3A*-null cells, in line with the loss of furin expression in these cells. Whether this reduced activity is a function of altered expression, maturation or stability of one or more furin-like proteases could not be established but, taken together, the above findings suggest that loss or inhibition of STT3A impacts convertase activity. Reduced activity or function may in turn compound the defects in receptor processing caused by their ER retention.

### Ectopically expressed PCSK5 is underglycosylated in *STT3A*-null cells and fails to rescue the receptor-processing defects

**Fig. 4.**
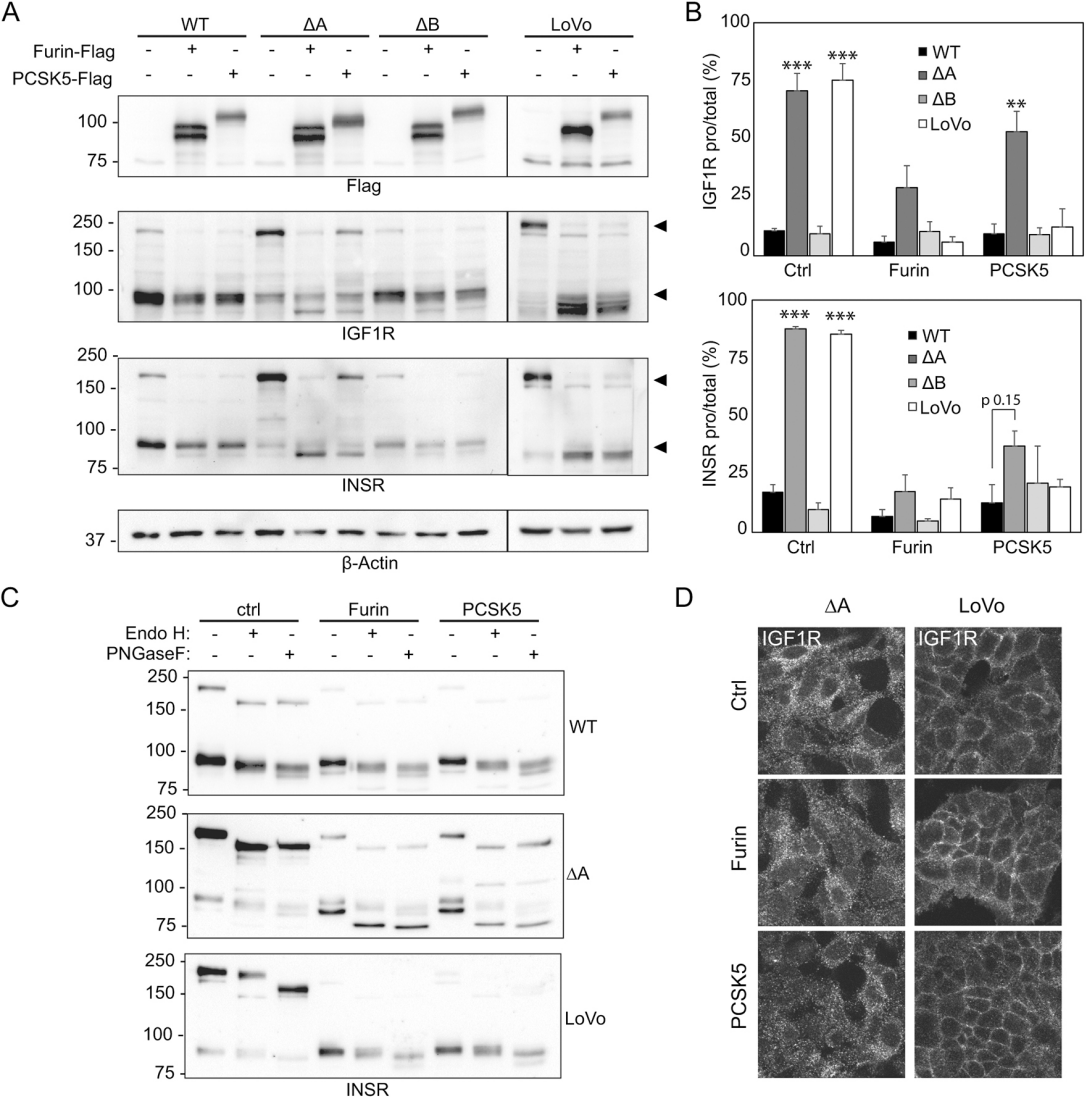
**Ectopically expressed PCSK5a is underglycosylated in *STT3A*-null cells and fails to rescue the receptor-processing defects*.*** (A) Representative western blots in HEK and LoVo cells transfected with Flag-tagged murine constructs for PCSK5a and furin. (B) Quantification of the level of pro-receptor relative to the total amount of receptor (pro-receptor+mature receptor). Average ratios from three (IGF-1R) or four (INSR) separate experiments are shown; error bars represent s.e.m. One-way ANOVA analysis was used to determine statistical significance. **P<0.01, ***P<0.001. (C) INSR western blot of PCSK5a- or furin-transfected HEK and LoVo cells, followed by Endo-H_f_- or PNGase-F treatment of the cell lysates. (D) IGF-1R immunostaining of PCSK5a- and furin-transfected and untransfected *STT3A*-null and LoVo cells.

We attempted to look at endogenous levels and electrophoretic mobility of the two main convertases responsible for receptor processing but were unsuccessful due to non-reactive commercial antibodies and/or the inherent instability of these proteases following zymogen activation. To overcome this limitation, we chose instead to ectopically express FLAG-tagged forms of these proteases in the cell lines studied. Expression clones of murine furin and PCSK5a were obtained and transfected in the cells followed by western blot for the tagged proteases, INSR and IGF-1R to determine whether either protease could restore normal receptor maturation. The murine and human convertases are highly conserved with identical numbers and position of their N-glycan sites. As shown in Fig. 4A, the tagged convertases are readily detected in all four transfected cell lines, although the electrophoretic mobility of PCSK5a was clearly increased in the *STT3A*-null cells. In contrast, furin did not appear to be altered in the *STT3A* mutant cells. Ectopic expression of both convertases in WT HEK cells increased processing of the small amount of receptor pro-forms. In *STT3A*-null cells, furin expression resulted in robust rescue of both the INSR- and IGF-1R-processing defect, whereas only minimal rescue was observed following PCSK5a introduction. The results are quantified in Fig. 4B. We then analyzed whether rescue of INSR processing by expression of tagged furin or PCSK5a resulted in a change in the localization of the pro- and mature forms of the receptor. After furin and PCSK5a introduction, the upper portion of mature INSR in *STT3A*-null cells is similar in its glycosidase sensitivity to what was observed in untransfected cells. However, there is another fraction of the mature form of the receptor (the lower portion) that remains Endo-H sensitive (Fig. 4C). We interpret this finding to reflect the processing of INSR in the early secretory pathway by the convertases but failure of the mature form of the receptor to reach the medial Golgi compartment and become Endo-H sensitive. The action of furin on INSR in the early secretory pathway was demonstrated in prior studies showing processing of mutant receptors in the ER despite the primary localization of furin in the trans-Golgi (Bass et al., 2000). As with the western blot results on INSR, we observed nearly all the IGF-1R staining inside the cells following convertase introduction into *STT3A*-null cells despite the efficient rescue of processing by furin and partial restoration by PCSK5a. Interestingly, the intracellular convertases are able to rescue receptor processing in LoVo cells even though much of the pro-form of IGF-1R can be found at the cell surface (Fig. 4D). One possible explanation for this finding is that newly synthesized receptor in the ER is processed in this compartment and eventually replaces the surface-localized pro-form receptor following the 48 h post-transfection period. The IGF-1R pro-form is also found inside LoVo cells, and may provide a pool of protein accessible to the overexpressed convertases in the ER.

### Lack of rescue by PCSK5a in *STT3A*-null cells does not arise due to differences in localization

**Fig. 5.**
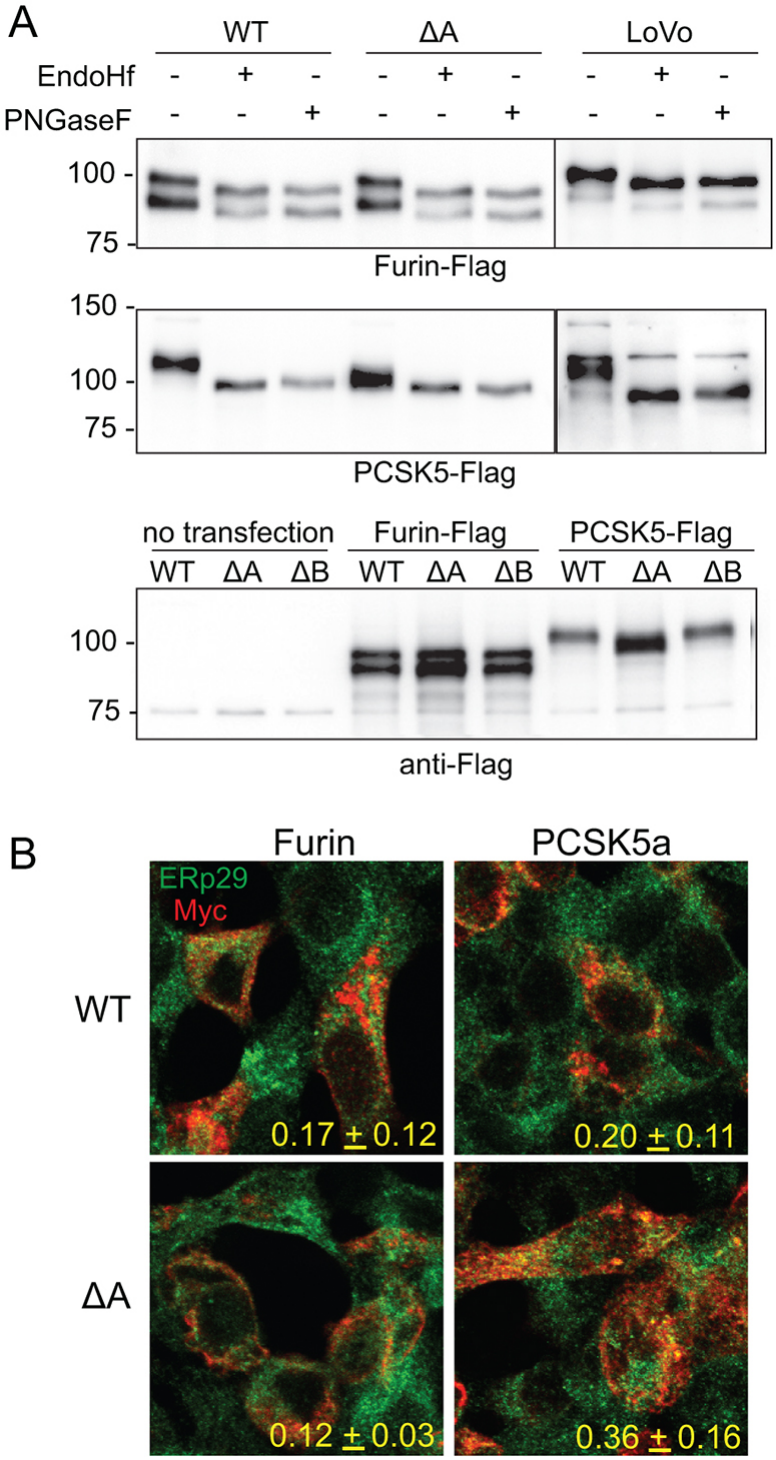
**Robust decreases in N-glycan occupancy on PCSK5a but not furin observed in *STT3A*-null cells.** (A) Representative western blot of three separate experiments of HEK WT and *STT3A*-null (ΔA) cells transfected with tagged murine constructs for PCSK5 and furin in untreated, Endo-H_f_- and PNGase-F-treated cell lysates. Lower panel gives a side-by-side comparison of the electrophoretic mobility of transfected furin and PCSK5 in the three HEK cell lines. (B) Myc and ERp29 co-immunostaining of PCSK5- and furin-transfected HEK WT and *STT3A-*null cells. Representative images are shown (*n*=3). Pearson colocalization coefficients were calculated from 15 different cell images from two different fields in the three separate experiments. Means±s.d. are shown.

To more closely examine the underglycosylation of the convertases, cells were again transfected with expression constructs for furin and PCSK5a, and cell lysates analyzed by western blot following Endo-H or PNGase-F treatment. The results in Fig. 5A clearly demonstrate the loss of N-glycans on PCSK5a when expressed in *STT3A*-null cells (shown most clearly in the lower panel where the different lines are compared side by side). The expressed enzyme detected in these cells runs as a heterogeneous band from 100-110 kDa and appears to contain some protein that is likely fully deglycosylated. Furin behaved similarly following transfection in all three lines with regard to Endo-H sensitivity, indicating that the convertase exists in a pre-Golgi compartment. We cannot rule out that the two N-glycans of furin remain Endo-H sensitive despite their transit through the Golgi. The Endo-H sensitivity of expressed PCSK5a also suggests that the protein largely remains in the ER or early Golgi. WT and *STT3A*-null cells expressing tagged-PCSK5a and furin were immunostained with antibodies to the Myc tag and the ER marker ERp29 (Fig. 5B). The results show that the convertases have some overlap with ERp29 but also exist in other non-Golgi compartments. The highest degree of colocalization was observed between ERp29 and PCSK5a in the *STT3A*-null cells. It is not clear whether overexpression of the tagged proteins saturated the ability of specific intracellular chaperones or binding proteins to carry the convertases to the trans-Golgi network or whether overexpression impairs the initial steps in convertase maturation. Nonetheless, PCSK5a rescues receptor processing in WT but not completely in *STT3A*-null cells, despite having similar localization and Endo-H sensitivity of their N-glycans. This difference argues that the loss of N-glycan occupancy likely alters the activity or zymogen activation of PCSK5a. It is possible, however, that the modest difference in localization between PCSK5a and furin in the *STT3A*-null cells is also a contributing factor.

### NGI-1 treatment of transfected HEK cells results in receptor-processing defects and significant underglycosylation of PCSK5

**Fig. 6.**
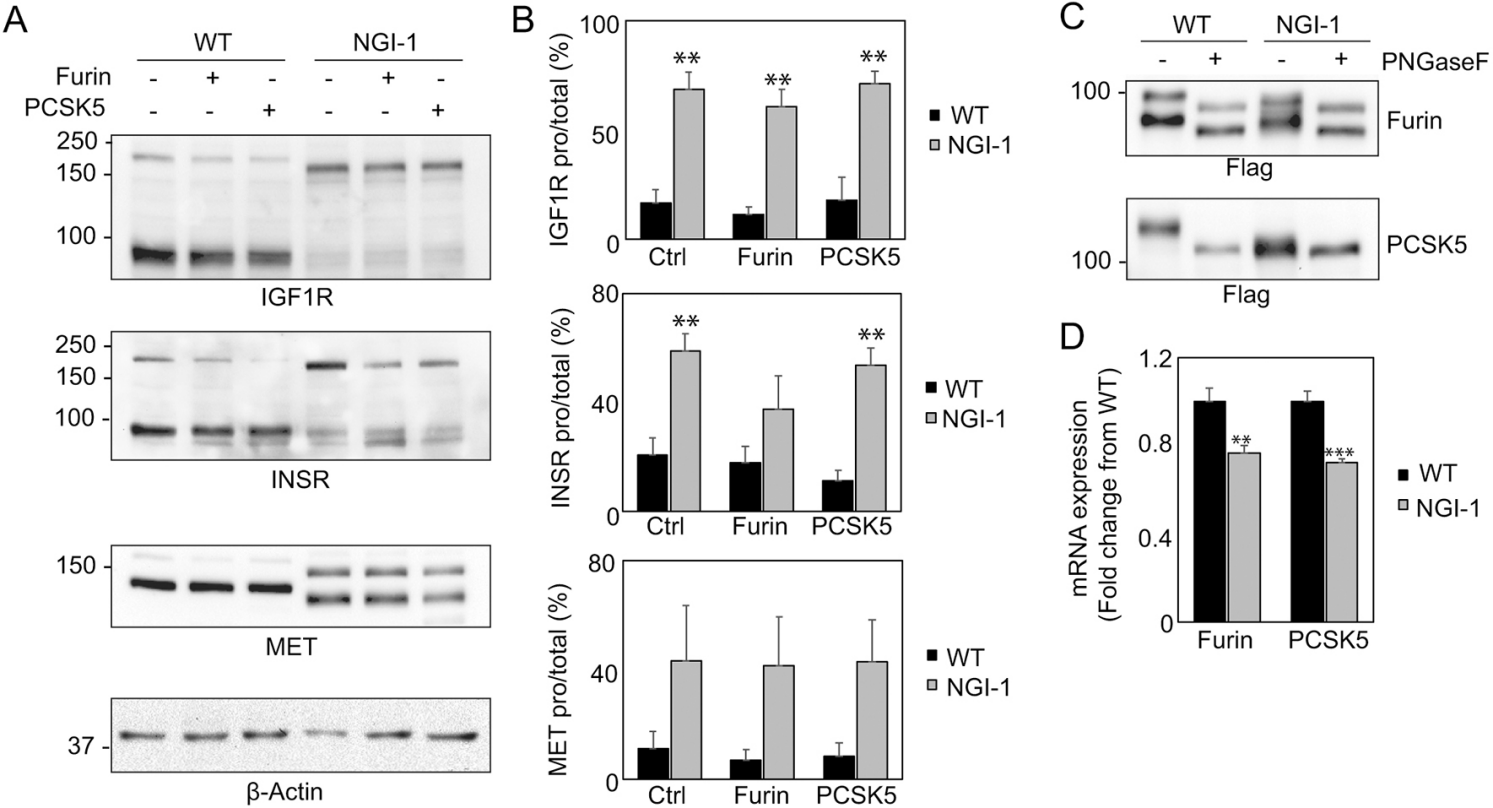
**NGI-1 treatment of transfected HEK cells results in underglycosylation of both furin and PCSK5, leading to more penetrant receptor-processing defects.** (A) Representative western blots of HEK WT and 10 µM NGI-1-treated cells transfected with Flag-tagged murine constructs for PCSK5a and furin (*n*=3). (B) Quantification of the level of pro-receptor relative to the total amount of receptor (pro-receptor+mature receptor) of three biological replicates. Error bars represent s.e.m. (C) Representative western blot from three separate experiments of HEK WT and NGI-1-treated cells transfected with Flag-tagged murine constructs for PCSK5a and furin in untreated and PNGase-F-treated cell lysates. (D) Quantitative PCR analysis of the transcript abundance of *PCSK5a* and furin in HEK WT and NGI-1-treated cells. **P<0.01, ***P<0.001.

The results above suggest that aberrant PCSK5 glycosylation in *STT3A*-null cells may contribute to IGF-1R- and INSR-processing defects. We next investigated whether treatment of WT HEK cells with NGI-1 would result in a similar effect on both convertase glycosylation and receptor processing. NGI-1 causes sequon skipping by the OST and reduces glycosylation of both STT3A- and STT3B-dependent sites (Rinis et al., 2018). Western blots of IGF-1R and INSR in either furin- or PCSK5a-transfected WT and NGI-1-treated cells shows a receptor-processing defect for both receptors (Fig. 6A). Unlike in *STT3A*-null cells, this processing defect is also observed with the Met receptor, likely pointing to the more robust effects of NGI-1 compared to the *STT3A* mutant. Expression of PCSK5a in NGI-treated cells failed to rescue the processing of INSR or IGF-1R and, interestingly, furin was only able to weakly restore processing in these treated cells (Fig. 6A,B). Western blot analysis of the two expressed convertases in NGI-1-treated cells shows a profound loss of N-glycosylation on PCSK5a but also increased heterogeneity on furin that was not detected in the *STT3A*-null cells (Fig. 6C). The effects on receptor processing do not arise from NGI-1-induced changes in convertase expression (Fig. 6D). These data suggest that PCSK5a and furin activity are sensitive to loss of N-glycosylation following acute OST inhibition with NGI-1 and show that pharmacological inhibition of the OST also disrupts convertase glycosylation and activity, which contributes to processing defects on both IGF-1R and INSR as well as Met receptor.

### Loss of specific N-glycans on PCSK5 and furin does not alter activity and ability to rescue receptor processing in the ER

**Fig. 7.**
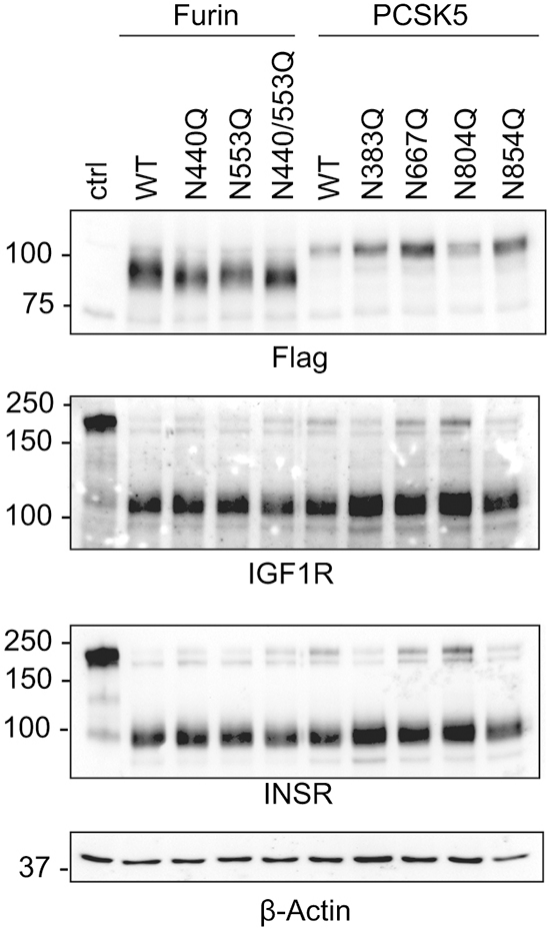
**Site-directed mutagenesis in PCSKa N-glycan sites does not affect rescue in LoVo cells.** Site-directed mutagenesis was done on four N-glycan sites of PCSK5a and two furin N-glycan sites. Representative Flag, INSR and IGF-1R western blots of LoVo cells transfected with WT or N-glycan-site-mutated constructs for PCSK5a and furin. β-actin was used as a loading control. Blots are representative of three individual transfections.

We next investigated whether removal of specific N-glycan sequons on both PCSK5a and furin would impact their activity and ability to restore receptor processing in the ER. Single N-sequon mutants for the two convertases were generated by site-directed mutagenesis, expressed in LoVo cells, and processing of both INSR and IGF-1R was analyzed by western blot (Fig. 7). The anti-FLAG blots showed the predicted increases in electrophoretic mobility following loss of the N-glycan sites on each convertase. Surprisingly, the overexpressed N-glycan mutants of each convertase are still able to rescue receptor processing, even the double N-glycan mutant of furin. For PCSK5a, it is likely that deletion of multiple N-glycan sites is required to recapitulate the findings noted after expression of PCSK5a in *STT3A*-null cells, based on the extent of the effects on this enzyme in both *STT3A*-null and NGI-treated WT cells. Another possibility is that loss of single N-glycan sites does affect activity but that overexpression of these proteins results in sufficient residual activity for receptor processing to still take place. Lastly, we cannot rule out that glycosylation defects on other ER glycoproteins in the *STT3A*-null and NGI-treated WT cells contributes to the failure of transfected PCSK5a to rescue receptor processing.

## DISCUSSION

Identifying how a reduction in the global N-glycosylation machinery of the cell impacts the function of specific glycoproteins remains a significant challenge towards defining the pathogenesis of CDGs. This challenge is embodied by the fact that multiple glycoproteins may lose one or more N-glycans in the context of many CDGs, but whether this loss is functionally relevant is not always apparent. The analysis of individual proteins is therefore warranted but choosing which proteins to focus on adds another layer of complexity. In this study, we uncovered a defect in the processing of two receptor tyrosine kinases in cells lacking the OST subunit STT3A that arises due to loss of N-glycans on the receptor themselves as well as altered glycosylation and activity of the proprotein convertase PCSK5a. All of these proteins are highly glycosylated, with INSR and IGF-1R bearing 19 and 11 potential N-glycan sites, respectively, and PCSK5a and furin bearing 5 and 2 potential sites, respectively. SEEL labeling of the WT and STT3 mutant HEK cell lines proved useful in identifying a significant reduction in both INSR and IGF-1R at the surface of the *STT3A*-null cells, allowing us to prioritize targets that exhibit functional consequences of decreased N-glycosylation – in this case, reduced cell surface localization. Western blot analysis of these receptors revealed near-normal steady-state levels, indicating that the processing defect in these receptors is associated with their failure to reach the cell surface. When OST function is more broadly and acutely inhibited using NGI-1, the receptor-processing defects encompass additional receptors such as Met, reflecting NGI-1-dependent defects in Met-receptor glycosylation and possible effects on multiple proprotein convertases.

Loss of N-glycosylation has been associated with defects in the activation of other proprotein convertases such as PC1/3 and SKI-1 (Zandberg et al., 2011), supporting a role for specific N-glycans in zymogen activation. The loss of activity following a reduction in N-glycan occupancy in the *STT3A*-null cells and NGI-1-treated cells is consistent with our measurement of global convertase activity (Fig. 3F). More substantial effects on activity may in fact be masked by the presence of another furin-like proprotein convertase that can utilize the furin substrate. We were unfortunately not able to monitor the glycosylation status of endogenous convertases to determine the extent of their underglycosylation in the *STT3A*-null cells. Nonetheless, our data align with a model whereby STT3A-dependent effects on INSR and IGF-1R as well as PCSK5a cause ER retention of the receptors and limit their processing in the ER when PCSK5a is overexpressed. It is interesting to note that several other glycoproteins with lower surface abundance in the *STT3A*-null cells, such as many α-integrins, are known substrates for PCSK5a (Lissitzky et al., 2000; Bergeron et al., 2003; Stawowy et al., 2004), highlighting other examples where combined effects on substrates and their convertases may be operational. One area of future investigation will be to determine whether reduction of other glycoproteins at the cell surface is due to direct effects on glycosylation on the glycoprotein, secondary effects on convertase function or a combination of the two.

The present findings shed important new light on the pathogenesis of CDGs associated with OST defects. The recently described STT3A-CDG manifests with microcephaly, developmental delay, seizures, intellectual disability and gastrointestinal symptoms (Shrimal et al., 2013a). These symptoms show significant overlap with the diseases caused by mutations in INSR or IGF-1R, two receptor tyrosine kinases that we have now shown to be underglycosylated when STT3A function is compromised. Mutations in both of these receptor tyrosine kinases are very rare, with IGF-1R mutation being found almost exclusively as a heterozygous mutation (Kruis et al., 2010; Wallborn et al., 2010; Labarta et al., 2013). Patients with IGF-1R mutation show growth retardation, microcephaly, feeding problems and mild intellectual impairment (Ester et al., 2009; Walenkamp et al., 2013). Mutations in INSR lead to Donohue syndrome, a rare autosomal dominant disease that is hallmarked by low body weight, microcephaly, muscle wasting and excessive thick skin (Kirkwood et al., 2018). The similarity in symptoms between INSR or IGF-1R mutation and STT3-CDG symptoms indicate a possible role for these proteins in the developmental pathogenesis of STT3A-CDG.

In the case of the receptor tyrosine kinases studied in this paper, the loss of surface localization is caused in part by the reduction of N-glycosylation on the receptors themselves. Nonetheless, the clear underglycosylation of PCSK5a and its failure to rescue processing when introduced in *STT3A*-null cells highlights an intriguing consideration for CDG pathogenesis: some glycoproteins may be indirectly affected by enzymes that are highly sensitive to abnormal glycosylation. Indeed, due to its five N-linked glycosylation sites, PCSK5a may serve as a sensor for abnormal glycosylation that can rapidly reduce cellular protease activity. Likewise, there may be cases where glycosylation of certain cell surface glycoproteins is altered but without a functional consequence. This complexity increases the challenge of unraveling pathogenic cascades in disorders where glycosylation is globally disrupted and a wide range of glycoprotein substrates are impacted. Studies to more fully describe how inhibition of N-glycosylation affects the spectrum of proprotein convertases in different tissues are ongoing and may lead to the identification of more sensitive cell surface glycoproteins.

IGF-1R and INSR are homologous members of the receptor tyrosine kinase superfamily of transmembrane glycoproteins, are widely expressed in normal tissues, and are expressed at high levels in malignant tumors. IGF-1R-stimulated downstream signaling, in particular, has become a target for inhibition by small molecules or receptor-specific antibodies in multiple tumor types. These approaches for targeting IGF-1R are based upon the development of successful therapies to other receptor tyrosine kinases such as EGFR and ErBB2 (Chon et al., 2006; Mishra et al., 2018). PCSK5 expression is a predictor for tumor response to the EGFR tyrosine kinase inhibitor erlotinib (Garnett et al., 2012). However, EGFR is not synthesized as a pro-receptor that requires cleavage to the mature form in the ER, suggesting that PCSK5 may have diverse contributions to the function of receptor tyrosine kinase signaling networks and that ‘upstream’ targeting of IGF-1R and EGFR maturation through blockade of protein convertases is an exciting area for future investigation. Our data suggest that PCSK5a, in the absence of other co-expressed protein convertases, would be a rationale target for blocking IGF-1R maturation and function. Our work indicates that the effects of abnormal glycosylation on INSR and IGF-1R are driven largely by abnormal convertase glycosylation, a distinct and previously unrecognized mechanism for receptor tyrosine kinase regulation by the OST. In addition, the blockade of IGF-1R processing by small-molecule inhibition of the OST with NGI-1 suggests that tumor therapy strategies that disrupt N-linked glycosylation could potently reduce IGF-1R signaling. In light of its effects on key oncogenic receptors, NGI-1 represents an attractive cancer chemotherapeutic candidate.

In summary, we demonstrate that selective blockade of N-glycosylation in *STT3A*-null cells reduces the maturation and cell surface localization of two receptor tyrosine kinases, INSR and IGF-1R. We also uncover PCSK5a as a substrate for STT3A and show that loss of glycosylation on this convertase reduces its activity and ability to rescue receptor processing when introduced into *STT3A*-null cells. This work further highlights the ability of chemical biology approaches such as SEEL to foster the identification of sensitive glycoproteins in the context of CDGs and elucidate the functional consequences of modulating N-linked glycosylation.

## MATERIALS AND METHODS

### Reagents

Recombinant rat α-(2,6)-sialyltransferase (ST6Gal1) was prepared as published previously (Meng et al., 2013). Biotinylated CMP-sialic acid was synthesized as previously reported (Sun et al., 2016). *Vibrio cholerae* neuraminidase (type II) (N6514) and Protein G Sepharose Fast Flow Beads (P3296) were purchased from Sigma-Aldrich. FastAP Thermosensitive Alkaline Phosphatase (EF0651), Protease Inhibitor Mini Tablets (88666) and compatible Silver Stain for Mass Spectrometry (24600) were purchased from Thermo Scientific. IgG fraction monoclonal mouse anti-biotin with and without HRP conjugation were purchased from Jackson ImmunoResearch Laboratories (200-032-211 and 200-002-011, 1:1000). Rabbit anti-IGF-1R-receptor β (Cell Signaling Technology, 9750, 1:250), rabbit anti-INSR β (Cell Signaling Technology, 3025, 1:1000), rabbit anti-furin (PA1-062) and rabbit anti-ERp29 (both Thermo Fisher Scientific, 1:1000), mouse anti-β-actin (Abcam, ab20272, 1:2000), rabbit anti-Met (Cell Signaling Technology, D1C2, 1:1000), mouse anti-calnexin (Thermo Fisher, AF18, cat. # MA3-027, 1:1000), mouse anti-ERGIC-53 (Axxora, ALX-804-602, 1:200) and rabbit anti-Flag (Sigma-Aldrich, F7425, 1:1000) were used. Fluorophore or HRP-conjugated anti-mouse or anti-rabbit secondary antibodies were used as appropriate for the corresponding primary antibody. HRP-labeled secondary antibodies were purchased from GE Healthcare. Goat anti-Myc was from Santa Cruz Biotechnology. Fluorescent secondaries used were purchased from Invitrogen Molecular Probes (Thermo Fisher Scientific). NGI-1 was prepared as previously described (Lopez-Sambrooks et al., 2016).

### Cell culture and transfections

*STT3A*-null (ΔA) and *STT3B*-null (ΔB) HEK293 cells were generated using the CRISPR/Cas9 genome-editing system as described (Cherepanova and Gilmore, 2016). Cells were cultured in 10 cm^2^ dishes at 37°C in DMEM with 4.5 g/l L-glutamine (Lonza, 12-604F), containing 10% fetal bovine serum (VWR, 1500-500), penicillin (100 IU/ml) and streptomycin (100 μg/ml) (Corning, 30-001-CI). LoVo human colon carcinoma cells were cultured in Ham's F12 medium with L-glutamine (Lonza, 12-615F), containing 10% fetal bovine serum (VWR, 1500-500), penicillin (100 IU/ml) and streptomycin (100 μg/ml) (Corning, 30-001-CI). Cells were transiently transfected with murine Flag-tagged Furin (Origene, MR210693) or Flag-tagged PCSK5 (Origene, MR222003) using Lipofectamine™ 2000 Transfection Reagent (Thermo Fisher, 11668030). Cells were collected 48 h after transfection.

### One-step SEEL labeling and proteomics

For labeling, cells were dissociated from the dish using Dulbecco's phosphate-buffered saline (DPBS) and transferred to Eppendorf tubes. Cell surface labeling was performed in serum-free DMEM with 42 μg/ml ST6Gal1, 34 μM CPM-Sia-C5-biotin, 13.3 μg/ml BSA, 13.3 μg/ml alkaline phosphatase and 2 μl *Arthrobacter ureafaciens* (AU) neuraminidase per reaction, with a reaction volume of 350 μl per tube for 2 h at 37°C. Lysis of labeled cells was done using RIPA buffer, followed by immunoprecipitation of 1 mg lysate using anti-biotin antibody. SDS-PAGE was done, followed by silver stain and in-gel digestion of proteins, as described previously (Sun et al., 2016). The resulting spectral count data were normalized by molecular mass and the average fold change (shown in log2 values) was calculated for the two replicates. Heat maps are shown for those that changed by more than 2-fold (log2 values <−1 or >+1) in *STT3A*- or *STT3B*-null cells. The comparative values for NGI-1-treated cells is also shown. In cases such as INSR, where no counts were detected in one sample, we assigned that protein a single spectral count in order to provide an estimated abundance.

### Western blotting and immunoprecipitation

Cells were washed with DPBS and then lysed on ice in RIPA buffer (50 mM Tris pH 8.0, 150 mM NaCl, 1% NP40, 0.1% SDS, 0.5% sodium deoxycholate) supplemented with protease inhibitor mixture. Protein concentrations were determined using the Micro BCA Protein Assay Kit (Thermo Scientific). Biotin immunoprecipitation with protein G beads was performed as reported previously (Yu et al., 2016). Proteins were separated by SDS-PAGE and transferred to 0.45 μm nitrocellulose membranes. Immunoreactive bands were identified with Clarity Western ECL substrate (Bio-Rad) and imaged and quantified using the Bio-Rad ChemiDoc MP Imaging System and Bio-Rad Image Lab software.

### IGF-1R stimulation

Cells were plated for 48 h prior to incubation with various doses of recombinant IGF-1 (source) for 2 h. Following activation, cells were washed with DPBS and then cells lysed on ice in RIPA buffer (50 mM Tris pH 8.0, 150 mM NaCl, 1% NP40, 0.1% SDS, 0.5% sodium deoxycholate) supplemented with protease inhibitor mixture and phosphatase inhibitors. Lysates were resolved by SDS-PAGE as described above and western blot analysis performed using an anti-phospho-IGF-1R antibody (Cell Signaling).

### Quantitative RT-PCR (qRT-PCR)

For total RNA isolation, the Qiagen RNeasy Plus Kit (74134) was used. cDNA synthesis was performed using the qScript cDNA synthesis kit with 500 ng RNA input (Quanta Biosciences, 95161). qRT-PCRs were performed using PerfeCTa SYBR Green FastMix (Quanta, 95072-250) on a Q-Tower^3^ (Analytik Jena) and data analysis was done using qPCRSoft 3.2 software. cDNA was checked for genomic DNA (gDNA) contamination by including control samples without reverse transcription. Ribosomal protein L4 (*rpl4*) was used as a normalization control.

### PCSK activity assay

PCSK activity was determined using a Boc-RVRR-AMC fluorogenic substrate (Enzo, ALX-260-040). The assay was optimized and fluorescent activity measured at Ex 360-380 nm, Em: 440-460 nm 60 min after addition of substrate. Substrate specificity was confirmed by addition of a PCSK inhibitor (Enzo, ALX-260-022), which completely blocked fluorescent activity.

### Confocal immunofluorescence microscopy

HEK WT, HEK *STT3A*-null, HEK *STT3B*-null and LoVo cells were plated on coverslips. When transfections were done, cells were transfected 24 h after plating and staining performed 48 h after transfection. For experiments without transfection, staining was done 24 h after plating. Immunofluorescence staining was performed according to the manufacturer's instructions (Cell Signaling Technology). Images were acquired on an Olympus FV1000 laser-scanning microscope outfitted with a 60× oil objective.

### Site-directed mutagenesis

Mutagenesis of PCSK5 and furin N-glycosylation sites was done using the QuikChange II Site-Directed Mutagenesis Kit (Agilent Technologies). Primers were designed to change Asn (N) sites to Gln (Q) according to kit instructions. A list of primers used can be found in Table S1. Site-directed mutagenesis by PCR was done according the manufacturer's protocol and mutagenesis confirmed by sequence analysis.

### Statistical analyses

Results are expressed as mean±s.e.m. Statistical analyses were performed using Graphpad Prism software. For paired comparisons of two groups, a paired samples *t*-test was performed (Fig. 6B,D). For other parametric data, a one-way ANOVA was performed, followed by Tukey's (Fig. 4B) or Dunnet's multiple comparison tests (Figs 2B and 3A,B). A value of *P*<0.05 was considered statistically significant. *P<0.05, **P<0.01, ***P<0.001. Pearson coefficients for the co-stains in Fig. 5B were calculated offline using SlideBook™ Imaging Software (Intelligent Imaging Innovations).

## Supplementary Material

Supplementary information
