# Supplementary material for: Selective inhibition of N-linked glycosylation impairs receptor tyrosine kinase processing
Source: Dis Model Mech. 2019 Jun 5;12(6):dmm039602. doi: 10.1242/dmm.039602 (PMC6602306; doi:10.1242/dmm.039602)
Supplement: Supplementary information [file dmm-12-039602-s1.pdf]

Table S1.

Click here to Download Table S1

Disease Models & Mechanisms • Supplementary information
